# Is There a “Window of Opportunity” for Flexibility Development in Youth? A Systematic Review with Meta-analysis

**DOI:** 10.1186/s40798-022-00476-1

**Published:** 2022-07-06

**Authors:** Olyvia Donti, Andreas Konrad, Ioli Panidi, Petros C. Dinas, Gregory C. Bogdanis

**Affiliations:** 1 School of Physical Education and Sport Science, National and Kapodistrian University of Athens, Athens, Greece; 2 Institute of Human Movement Science, Sport and Health, University of Graz, Graz, Austria; 3 FAME Laboratory, Department of Physical Education and Sport Science, University of Thessaly, Volos, Greece

**Keywords:** Stretching, Children, Adolescents, Training

## Abstract

**Background:**

Flexibility is an important component of physical fitness for competitive and recreational athletes. It is generally suggested that flexibility training should start from childhood (6–11 years of age) to optimize joint range of motion (ROM) increases; however, evidence is limited and inconsistent.

**Objective:**

To examine whether there is a difference in the effect of stretching training on flexibility during childhood (6–11 years of age) and adolescence (12–18 years of age).

**Design:**

Systematic review and meta-analysis.

**Methods:**

We searched PubMed Central, Web of Science, Scopus, Embase, and SPORTDiscus, to conduct this systematic review. Randomized controlled trials and non-randomized controlled trials were eligible. No language and date of publication restrictions were applied. Risk of bias was assessed using Cochrane RoB2 and ROBINS-I tools. Meta-analyses were conducted via an inverse variance random-effects model. GRADE analysis was used to assess the methodological quality of the studies.

**Results:**

From the 2713 records retrieved 28 studies were included in the meta-analysis (*n* = 1936 participants). Risk of bias was low in 56.9% of all criteria. Confidence in cumulative evidence was moderate. We found that stretching was effective in increasing ROM in both children (SMD = 1.09; 95% CI = 0.77–1.41; *Z* = 6.65; *p* < 0.001; *I*^2^ = 79%) and adolescents (SMD = 0.90; 95% CI = 0.70–1.10; *Z* = 8.88; *p* < 0.001; *I*^2^ = 81%), with no differences between children and adolescents in ROM improvements (*p* = 0.32; *I*^2^ = 0%). However, when stretching volume load was considered, children exhibited greater increases in ROM with higher than lower stretching volumes (SMD = 1.21; 95% CI = 0.82–1.60; *Z* = 6.09; *p* < 0.001; *I*^2^ = 82% and SMD = 0.62; 95% CI = 0.29–0.95; *Z* = 3.65; *p* < 0.001; *I*^2^ = 0%, respectively; subgroup difference: *p* = 0.02; *I*^2^ = 80.5%), while adolescents responded equally to higher and lower stretching volume loads (SMD = 0.90; 95% CI = 0.47–1.33; *Z* = 4.08; *p* < 0.001; *I*^2^ = 83%, and SMD = 0.90; 95% CI = 0.69–1.12; *Z* = 8.18; *p* < 0.001; *I*^2^ = 79%, respectively; subgroup difference: *p* = 0.98; *I*^2^ = 0%).

**Conclusions:**

Systematic stretching training increases ROM during both childhood and adolescence. However, larger ROM gains may be induced in childhood than in adolescence when higher stretching volume loads are applied, while adolescents respond equally to high and low stretching volume loads.

*Registration:* INPLASY, registration number: INPLASY202190032; https://inplasy.com/inplasy-2021-9-0032/

**Supplementary Information:**

The online version contains supplementary material available at 10.1186/s40798-022-00476-1.

## Key Points

- Systematic stretching training increases ROM during both childhood and adolescence.
- Larger ROM gains may be induced in childhood than in adolescence when higher stretching volume loads are applied
- Adolescents respond equally to high and low stretching volume loads

## Background

Long-term athlete development models provide general frameworks to prepare children and adolescents for sports and a physically active lifestyle [1]. These models aim to align sport practice with growth, maturation, and early sport specialization and consider factors such as injury risk [2, 3] and the limitations of the existing training practice schedules [4]. Muscular strength and power, speed, agility, mobility, and flexibility are central fitness components in all the long-term athlete development models [3, 5, 6]. Most long-term athlete development models encourage participation in mobility and flexibility training from a very young age (45 years), with an underlying assumption that flexibility can be enhanced more with early training [7, 8].

Flexibility is an important component of physical fitness for competitive and recreational athletes [9] and a performance determinant in sports requiring the ability to move comfortably through a large range of motion (ROM) [10]. Flexibility is defined as the ROM in a joint or series of joints [9] and from a functional perspective represents the ability to move comfortably without constraints or pain through a full ROM [11]. The importance of flexibility in children and adolescents is task and sport specific [10]. For example, in gymnastics the athlete executes skills assuming extreme body positions [12, 13], while in other sports, a large ROM is utilized to enhance the mechanical effectiveness of a task [14, 15]. For example, in throwing activities an enhanced joint ROM can increase the distance over which muscle force is applied or absorbed thus allowing the athlete to generate a higher power output [15, 16]. In sports such as gymnastics [12, 13] and throwing [16], increased hip and shoulder ROM are typically associated with higher performance level. There is also evidence suggesting that decreased joint ROM is a risk factor for injury in young athletes [17, 18]. For example, adolescent swimmers with limited ROM were found to have a 3.6 times higher risk of developing shoulder pain than swimmers with normal ROM [17].

Despite its importance, flexibility is a largely under-researched area of study within the pediatric populations [15]. It has been suggested that childhood is a key time period for flexibility development, with the age range of 6–11 years proposed as being a “window of opportunity” for flexibility development [8]. One possible mechanism for this is the increased pliability and reduced musculotendinous stiffness associated with childhood [19], which may enable greater ROM to be attained, and this may, in turn, render flexibility training more effective. For example, Kubo et al. [19] reported that the tendon structures in younger boys (10–11 years old) are more compliant than those in older boys (14–15 years old) and young men, although the association between muscle and tendon mechanical properties and joint ROM in children and adolescents has not been investigated. Furthermore, children and adolescents are generally more flexible than adults [20, 21], while joint ROM gradually diminishes with age [22]. However, research on flexibility training in youth is limited, and evidence regarding the existence of “windows of opportunity” for different motor skills development is controversial [23]. Previous long-term athletic development models did not suggest an appropriate period for flexibility development [7]. More recently, the Youth Physical Development Model [8] suggested that middle childhood (ages 6–11) may be an optimal time frame for flexibility and mobility training. According to the authors of this model, the rationale for this selection is that it incorporates a period that has previously been termed a “critical period” of flexibility development, which is supported mainly by empirical evidence [10, 24]. For example, in sports such as gymnastics and dance, children are submitted to extensive daily flexibility training schedules on the assumption that ROM gains may be maximized with early training [10]. On the other hand, the levels of flexibility tend to plateau or even decrease at the time of the adolescent growth spurt and into adulthood, especially in boys, thus lending support to the notion of a “window of opportunity” earlier in childhood, at least in boys [25].

Short-term stretching training improvements in joint ROM are usually attributed to increased stretch tolerance and/or are related to a decreased tissue resistance to stretch [9]. The loading characteristics of the stretching protocol are key elements for chronic joint ROM increases [26]. Past research in adults has reported that total stretch duration is more important for ROM enhancement than the duration of each stretching bout [27]. Cross-sectional studies in adults also reported that higher stretching volume load (i.e., the total duration of stretching applied over the intervention period) is a crucial factor for improvement in ROM [28]. However, evidence for the effects of stretching training on ROM improvement in children and adolescents is limited and, in many cases, contradictory [29]. Thus, although a “window of opportunity” for flexibility development has been widely suggested, there is only sparse evidence to verify its existence. Moreover, the effect of confounding variables such as the loading characteristics of the stretching protocols has not yet been collectively assessed. Therefore, the aim of this systematic review and meta-analysis was to examine whether there is a difference in the effect of stretching training on flexibility during childhood (6–11 years of age) and adolescence (12–18 years of age).

## Methods

### Study Design

This systematic review was conducted according to the Preferred Reporting Items for Systematic Reviews and Meta-Analyses (PRISMA) guidelines [30] (see Additional file 1: PRISMA checklist). The review was preregistered in the International Platform of Registered Systematic Review and Meta-analysis Protocols (INPLASY, registration number: INPLASY202190032; https://inplasy.com/inplasy-2021-9-0032/).

### Search and Selection Strategy

Five electronic databases were searched through, until March 2022 by two independent investigators (OD, IP): PubMed Central, Scopus, Web of Science, Embase and SPORTDiscus. No language and date restrictions were applied. The search was carried out in the field type “Title and abstract.” The topic was systematically searched using a Boolean search strategy with the operator “AND” and “OR.” The keywords with more than one word were enclosed in quotes. The keyword algorithm used in the selected databases can be found in Additional file 1. Additional records that were not picked up in systematic searches were identified through: (1) searching the reference lists of original studies and some related study reviews, (2) examining the reference citations and the researchers’ publications, (3) contacting by email the corresponding authors (if they were not defined, the first author was used), and (4) screening the researchers’ personal lists in ResearchGate and Google Scholar (first authors) [31, 32]. Based on our knowledge of the area, we also contributed additional studies which we had knowledge of but were not picked up in systematic searches. Two investigators (IP, AK) selected the eligible studies based on the eligibility criteria. In the case of a disagreement between the investigators, GCB and OD made the ultimate decision for the searching and selection procedures by majority consensus.

### Inclusion and Exclusion Criteria

We followed PICOS (Population, Intervention, Comparison, Outcome, Study Design) for selecting studies for inclusion. We included randomized controlled trials and non-randomized controlled trials (not randomized trials that include a comparison or control group). The included studies investigated the chronic effects (> 2 weeks) of static stretching in healthy (i.e., non-clinical) children (5–11 years old), and adolescents (12–18 years old). We included pupils, recreationally active, and trained participants. Studies also had to include an implementation of a static stretching intervention because evidence for other types of stretching (e.g., dynamic, ballistic, proprioceptive neuromuscular facilitation stretching, and nerve-directed stretching) is limited in children and adolescents and these types of stretching are not commonly used in physical education and sport settings in these age groups. Due to the limited evidence, we also decided to include only studies that examined lower limbs. The comparison conditions included pre- and post-stretching interventions in experimental and control conditions. Data regarding ROM maintenance following a detraining period were not included in the study. We excluded single group studies, studies without a control group, studies which had no clearly defined stretching protocol or a protocol also including a different stimulus (e.g., vibration or strength training). In addition, studies which focused on very small joints (e.g., fingers, toes), non-human studies, and in vitro studies were excluded. Retrospective studies, review papers, case reports, special communications, letters to the editor, invited commentaries, and conference papers were excluded. Related articles were included up to March 2022.

### Risk of Bias Assessment and Methodological Quality

IP and OD independently assessed the risk of bias of the included studies and any conflict was resolved through discussion with AK and PCD. The updated Cochrane Risk of Bias 2 (RoB2) and ROBINS-I tools were used for the randomized controlled trials and controlled trials without randomization, respectively. The updated Risk of Bias 2 (RoB2) Cochrane Library includes the following sources of bias: bias arising from the randomization process, bias due to deviations from intended interventions (effect of assignment to intervention and effect of adhering to intervention), bias due to missing outcome data, bias in the measurement of the outcome, and bias in selection of the reported result [33]. ROBINS-I includes the following bias domains: bias due to confounding, bias in selection of participants into the study, bias in classification of interventions, bias due to deviations from intended interventions, bias due to missing data, bias in measurement of outcomes, and bias in selection of the reported results [34].

### Confidence in the Cumulative Evidence

The Grading of Recommendations, Assessment, Development and Evaluations (GRADE) quality rating analysis was used to assess the quality of the outcomes. GRADE has four levels of evidence quality: very low, low, moderate, and high [35, 36]. For GRADE analysis, five evaluation components were adopted to lower quality (risk of bias, inconsistency of results, indirectness, imprecision, and publication bias) and three evaluation components to higher quality (large effect, dose–response, and confounding). All evaluation components were assessed independently by OD and IP and verified by GCB and PCD. The same authors estimated the overall quality and confidence in the cumulative evidence.

### Data Extraction

Three independent investigators (AK, IP, and OD) extracted the data from the included papers in the systematic review. The data extraction was supervised by two other investigators (PCD and GCB). We extracted data regarding: (a) author and year of publication, (b) type of publication (journal paper or grey literature), (c) study design (randomized controlled trial or controlled trial), (d) sample size in total, and for the experimental and control groups, (e) sex (males and females), (f) age (for the experimental and the control groups), (g) anthropometric characteristics (body mass, height), (h) participants’ physical activity level (e.g., recreationally active, athlete, or pupil), (i) the main outcome of the study, and (j) the means and standard deviations for outcome measures for both the experimental and the control groups. The term “Range of motion (ROM)” was used to indicate the linear or angular distance and direction a joint can move between the flexed position and the extended position [10]. The characteristics of the included studies can be found in Table 1. In addition, we extracted the characteristics of the stretching interventions, the joint, and muscle examined and the test used to assess ROM. Additional details regarding the stretching intervention characteristics (i.e., the duration of every stretching bout, the number of exercises, the number of sets, and the frequency of stretching training per week) were extracted and from these data, and we calculated the daily stretching duration (s) (the duration of each stretching bout × number of sets × number of exercises), the stretching duration per week (s) (the duration of the daily stretching × the number of stretching trainings per week), and the total duration of the stretching intervention (s) (the stretching duration per week × the number of weeks). These characteristics can be found in an open repository file (10.6084/m9.figshare.17104640).

**Table 1:** Characteristics and main outcomes of the included studies

Study	Study design	Participants -total (n)	Males	Females	SG (n)	CG (n)	Age (SG)	Age (CG)	Physical activity	Stretching volume (s)	Stretch Intensity	Cohen’s *d*	Main outcome
Azuma and Someya [48]	RCT	124	124	–	64	60	16.2 ± 0.8	16.2 ± 0.8	Soccer	3240	No pain	0.74–1.51	The SG showed significant higher values in heel–buttock distance, straight leg raise, hip rotation angles, and ankle dorsiflexion at 12 weeks relative to pre-intervention values. Positive effects were also found in injury rate. No increases were observed in the CG
Beccera-Fernadez et al. [49]	RCT	102		102	49	53	16–17	16–17	Students	1920	Stretch position was held gently	0.27	A physical education-based program for eight weeks significantly improved students’ hamstrings extensibility in the SG compared to the CG
Coledam et al. [29]	CT	58	29	29	14	15	8.6 ± 0.7	8.5 ± 0.5	Students	3840	NR	0.88 (boys)	Performance of stretching exercises during warm-up for 16 weeks significantly improved sit and reach score in the SG compared to the CG
					14	15	8.4 ± 0.5	8.6 ± 0.7	Students			1.26 (girls)	
de Baranda [50]	RCT	50	23	27	26	24	13.7 ± 0.4	13.7 ± 0.4	Students	16,740	NR	1.30–1.47	Significant improvements were found in straight leg raise in the SG compared with the CG after 31 weeks of stretching training
Donti et al. [43]	CT	77	–	77	57	20	9.3 ± 0.8	8.9 ± 0.6	Gymnasts	4050	POD	2.27 (intermittent stretching) 0.91 (continuous stretching)	ROM increased following an intermittent or a continuous static stretching program of 12 weeks in youth female gymnasts but remained unchanged in the control group. Intermittent stretching conferred a larger improvement compared to continuous
Donti et al. [44]	CT	30	–	30	19	11	9.8 ± 0.5	9.5 ± 0.8	Gymnasts	2430	POD	0.92 (intermittent stretching) 0.60 (continuous stretching)	Hip hyperextension ROM increased similarly following an intermittent or a continuous stretching protocol of 9 weeks. No increase in ROM was observed in the CG. Counter movement jump height after stretching was not affected by either stretching protocol
Hadjicharalambous [51]	RCT	23	23		11	12	16.1 ± 0.7	16.0 ± 0.6	Soccer	6144	Point of mild discomfort	1.48	Flexibility training for four weeks improved sit and reach score, 35 m sprint, agility and broad jump in adolescent soccer players more than soccer training alone
Hill and Najera [45]	CT	102	53	49	60	42	13–15	13–15	Students	3240	Tightness/no pain	0.60	A significant improvement was observed in the SG compared to CG in hamstring extensibility following 9 weeks of stretching in a physical education setting
Kamandulis et al. [52]	CT	229	107	122	62	58	15.1 ± 0.3	15.2 ± 0.5		200 800 3200		0.24 (boys) 0.18 (girls)	Static stretching performed once, four times, or in a set of four exercises repeated four times during physical education classes increased ROM in adolescent students; however, the higher volume stretching protocol induced greater improvements in ROM. No improvement was observed in the CG
					55		15.0 ± 0.4		Students	200 800 3200	NR	0.40 (boys) 0.58 (girls)	
					54		15.0 ± 0.4			200 800 3200		0.60 (boys) 0.81 (girls)	
Knapik et al. [53]	RCT	106	46	60	51	55	15.8 ± 1.3	16.3 ± 1.0	Basketball	50,400	NR	0.25 (boys) 0.53 (girls)	Athletes undergoing gastrocnemius stretching demonstrated significant increases in ankle dorsiflexion following 3 months of training and greater ankle dorsiflexion compared with controls at all time points
Mayorga-Vega et al. [57]	RCT	73	36	37	25	24	9.0 ± 0.2	9.0 ± 0.2	Students	3600	Gentle feeling of tightness Gentle feeling of tightness	0.09 (warm-up) 0.11 (cool down)	Students performing stretching either during warm-up or cool down for ten weeks had higher values in hamstrings extensibility than no-training students. No difference was observed between warm-up and cool down groups
					24		9.0 ± 0.2		Students	3600			
Mayorga-Vega et al. [58]	RCT	45	24	21	22	23	9.9 ± 0.3	9.9 ± 0.3	Students	3840	Tightness/no pain	0.28	A short-term (5 weeks) stretching intervention increased sit and reach score in elementary school children. No increase was observed in the CG
Mayorga Vega et al. [59]	RCT	45	26	19	22	23	10.9 ± 0.3	10.9 ± 0.3	Students	4800	Tightness/no pain	0.34	A stretching intervention during physical education classes significantly increased hamstring and lumbar extensibility in the SG compared to the CG
Mayorga-Vega et al. [54]	RCT	163	84	79	53	58	12.7 ± 0.7	12.6 ± 0.6	Students	1920	Tightness/no pain	0.23	Stretching performed once or twice a week during physical education class improved hamstrings extensibility in the two SG compared to CG. No differences were observed between the two SG
					52		12.7 ± 0.6		Students	3840		0.24	
Mayorga-Vega et al. [56]	RCT	89	43	46	44	45	8.5 ± 0.8	8.4 ± 0.6	Students	4320	Tightness/no pain	0.47	A physical education-based stretching intervention for 9 weeks improved more hamstrings extensibility in the SG compared to the CG
Mayorga-Vega et al. [55]	RCT	37	18	19	19	18	9.0	9.0	Students	5040	Tightness/no pain	0.71	A physical education stretching program for 34 weeks increased sit and reach score in the SG compared to the CG
Merino-Marban et al. [61]	RCT	45	26	19	23	22	5.9 ± 0.3	5.9 ± 0.3	Students	960	Tightness/no pain	0.45	An 8-week stretching intervention significantly increased the sit and reach score in the SG compared to the CG
Moreira et al. [62]	RCT	58	30	28	28	30	12.2 ± 1.8	11.9 ± 2.1	Students	360	Stretching as much as tolerable	0.52	A six weeks stretching program improved hamstrings extensibility in secondary school children. No improvement was observed in the CG
Nelson and Bandy [63]	RCT	45	45	–	21	24	16.2 ± 1.1	16.5 ± 1.0	Students	540	Gentle feeling of stretch	1.72	Six weeks of static stretching increased hamstrings extensibility in adolescent males compared to controls
Panidi et al. [64]	RCT	21	–	21	21	21	13.5 ± 1.4	13.5 ± 1.4	Volleyball	45,900	POD	3.48	High-volume static stretching of the plantar flexors was applied for 12 weeks to one leg with the contralateral leg as control. Ankle dorsiflexion increased in both legs with a greater increase in the stretched compared to control leg. A greater increase was also observed in jumping height in the stretched compared to the control leg
Piqueras-Rodríguez et al. [65]	RCT	42	42	–	21	21	12.3 ± 1.6	12.3 ± 1.8	Soccer	720	Tolerable stretch	0.17–0.99	Stretching increased straight leg raise, toe-touch and passive knee extension scores in the SG compared to CG after 8 weeks of training
Reid and McNair [68]	RCT	43	43	–	23	20	15.8 ± 1.1	15.7 ± 0.9	Students	2700	NR	1.45	Stretching intervention induced significant increases in passive knee extension range of motion, passive resistive force and stiffness in the SG while no differences were observed in the CG
Rodríguez et al. [66]	RCT	46	20	26	25	21	10.3 ± 0.3	10.3 ± 0.3	Students	19,200	Gentle feeling of stretch	0.28	Five minutes of stretching during physical education classes for a period of 32 weeks, significantly increased sit and reach score in elementary school children whereas the score decreased in the CG
		44	21	23	24	20	13.5 ± 0.7	13.5 ± 0.7	Students	19,200		0.93	
Sánchez Rivas et al. [67]	RCT	44	20	24	22	22	7.9 ± 0.4	7.9 ± 0.4	Students	3240	POD	0.30	A three-minute static passive hamstrings stretching program over nine weeks improved sit and reach score in the SG compared to CG
Santonja Medina et al. [60]	CT	63	NR	NR	25	18	10.3 ± 0.5	10.3 ± 0.3	Students	4960	Gentle feeling of stretch	0.90 (left leg) 0.93 (right leg)	Significant improvements in ROM were observed after a school term (9 months) of flexibility training in the two experimental groups compared to the control group that followed a standard physical education class
					20		10.5 ± 0.6		Students	9920		1.68 (left leg) 2.10 (right leg)	
Sermaxhaj et al. [46]	CT	24	24	–	12	12	13.8 ± 0.5	14.0 ± 0.4	Soccer	10,560	NR	0.40	Static stretching performed at the end of the training session for 4 months did not increase sit and reach score in the SG compared to the CG
Sermaxhaj et al. [47]	CT	68	68		10	10	12.0 ± 0.4	11.9 ± 0.5		8640		0.60	Following 16 weeks of stretching, only the SG of the cadet football players (15–17 years old) improved their sit and reach score compared with the CG
					12	12	13.8 ± 0.5	14.0 ± 0.4	Football	8640	Further possible bend	0.40	
					12	12	15.9 ± 0.6	15.6 ± 0.4		8640		0.60	
Vans Rensburg and Coetzee [69]	RCT	40		40	20	20	13–17	13–17	Students	2700	Point of tightness	2.74	After six weeks of static stretching, hamstrings extensibility increased in the SG compared with the CG
All Participants (n)		1936	975	898									
Participants ≤ 11 years (n)		652	242	347									
Participants ≥ 12 years (n)		1284	703	523									

ROM: Range of motion; Cohen’s d: *d*: values from pre- to post-intervention for the experimental groups; CT: controlled trial; RCT: randomized controlled trial; SG: stretching group; CG: control group; POD: point of discomfort; and NR: not reported

### Data Synthesis and Meta-analysis Methods

All the included studies in the systematic review provided data for the meta-analysis. We extracted pre- and post-intervention means and standard deviations. In the case of data being given in the form of a graph and in the case of missing data, the corresponding or first authors of the included studies were contacted via email, to retrieve these data. We have calculated the Δ scores of the means by subtracting the baseline values from the post-intervention values. The standard deviations for the Δ scores were calculated according to the following equation: $\sqrt {\left( {{\text{SD}}^{2} {\text{pre}} + {\text{SD}}^{2} {\text{post}}} \right){-}\left( {2 \times 0.70 \times {\text{SDpre}} \times {\text{SD post}}} \right) }$ [33]. This approach removed the bias acquired from the significant differences in baseline values that might have played a role in the post-intervention differences between the experimental and control groups. We conducted an inverse-variance, continuous, random-effects model meta-analysis using RevMan 5.3 software [34]. We tested the differences in ROM between an experimental (stretching group) and a control group (i.e., no stretching). Heterogeneity was tested using the *I*^2^ statistic [35]. *I*^2^ values indicate the degree of heterogeneity in the effects: 0–40% were not important, 30–60% moderate heterogeneity, 50–90% substantial heterogeneity, and 75–100% considerable heterogeneity [36]. A cutoff value of 75% was adopted as an index of considerable heterogeneity. In all the meta-analyses, we used the standardized mean differences due to the different scale measurements that the variables displayed [33]. We performed between group analyses, which included comparisons of age (children 5–11 years of age vs. adolescents 12–18 years of age) irrespective of the stretching protocol, and between group analyses which included comparisons between high and low stretching volume loads (< 3600 s vs. ≥ 3600 s) irrespective of age. Subgroups analyses were performed according to age groups, as follows: child participants (≤ 11 years of age) following either a lower (< 3600 s) or a higher stretching volume load protocol (≥ 3600 s), and adolescents (≥ 12 years of age) following either a lower (< 3600 s) or a higher stretching volume load protocol (≥ 3600 s). The age groups were selected based on evidence of age-related differences in growth [37, 38], motor skill competence, and health-related physical fitness [39, 40]. The cutoff value for the stretching volume load was determined by calculating the total stretching duration (in s) of 10 weeks of training, including three sessions per week, and performing in each session two sets of two exercises lasting 30 s each (< 3600 s). This duration was selected to reflect typical stretching training protocols in sports and school practice [41]. No comparisons between the athletic and non-athletic populations were performed because, in the studies involving primary or secondary school students, extracurricular activities (e.g., sport participation) were either not controlled for or not reported. In addition, no subgroup comparisons between male and female participants were conducted because the studies including both males and females reported collective values for both sexes. According to Hopkins et al. [42], we defined the effects for a standardized mean difference (SMD) of < 0.2, 0.2–0.6, 0.6–1.2, 1.2–2.0, 2.0–4.0, and > 4.0 as trivial, small, moderate, large, very large, and extremely large, respectively. An alpha level of 0.05 was defined for the statistical significance of all the tests, apart from heterogeneity (*p* < 0.10). Moreover, visual inspection of the funnel plot was applied to detect possible publication bias.

## Results

### Results of the Searching Procedure

**Fig. 1:**
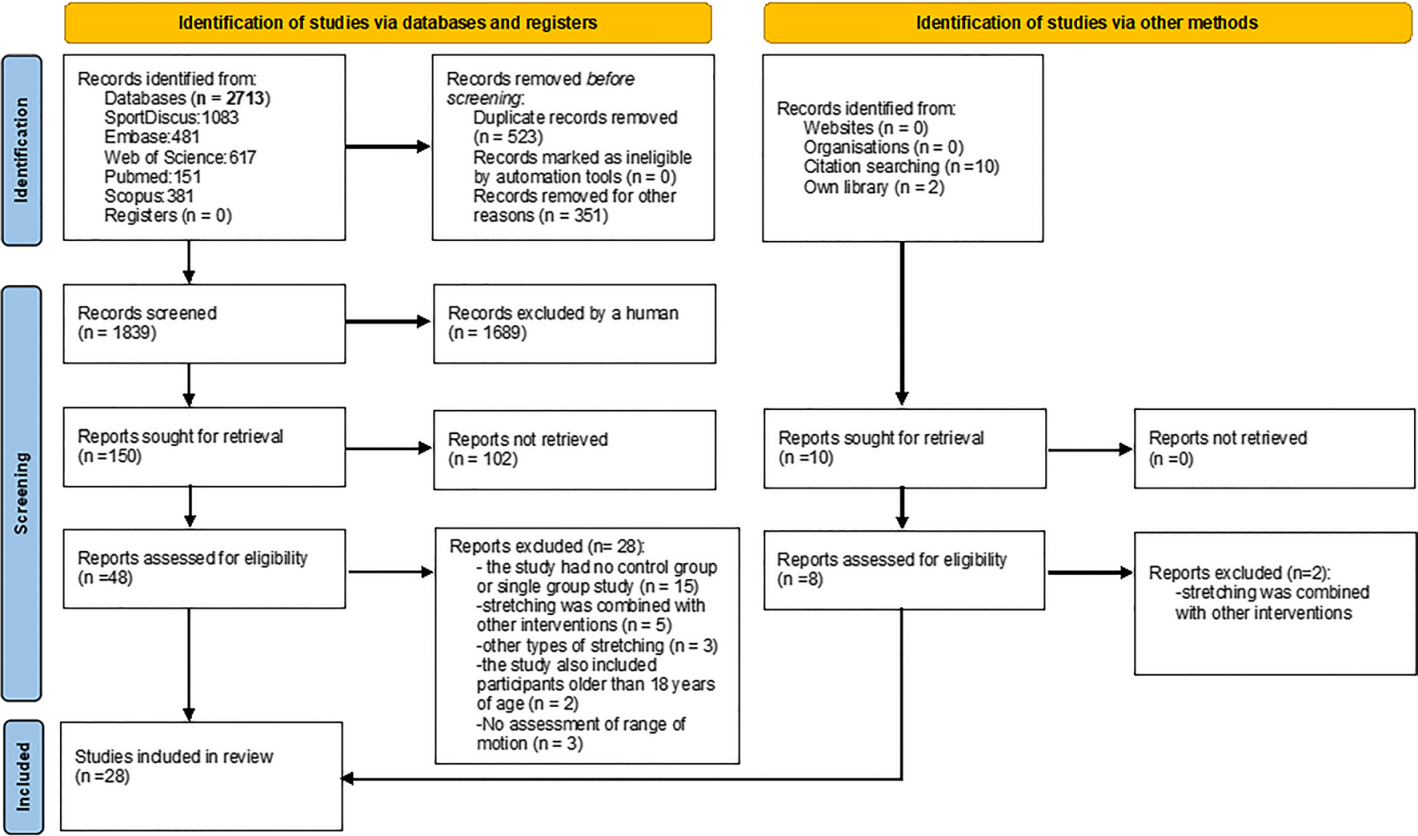
PRISMA flowchart illustrating different phases of the search and study selection [30]

The initial search procedure retrieved 2713 papers. After duplicates were removed (*n* = 523), 2190 papers remained for eligibility evaluation. From these 2190 papers, 163 were conference papers, one was a letter to the editor, 162 papers were reviews, 25 were published proceedings and 1791 were considered irrelevant because they examined adult or clinical populations, acute interventions, or interventions not relevant to the study purpose. Finally, 48 papers were found to be eligible for this study. We then checked the reference lists and citations of the eligible studies to determine whether additional studies were relevant. Following this additional search, 8 more relevant papers were identified, of which 6 papers were eligible. Also, two more papers were added from our own library. After the screening of the full texts of the 56 eligible papers, 28 papers were excluded for different reasons (i.e., the study had no control group, or the study included some other type of stretching or stretching was combined with other interventions such as vibration or strength training). Therefore, in total, 28 papers (54 entries) were included in this systematic review and were used in the meta-analysis. A flowchart of the search process is presented in Fig. 1.

### Characteristics of the Included Studies

The 28 eligible studies in this systematic review and meta-analysis were published between 2004 and 2021 and involved 1936 participants (975 males). In total, 652 participants were between 5 and 11 years of age and 1284 participants were between 12 and 18 years (mean age: 9.3 ± 1.4 years vs. 14.0 ± 2.7 years, respectively). The characteristics of the participants can be found in Table 1. Out of the 28 eligible studies, six were controlled trials (CTs) [29, 43–47], and 22 were randomized controlled trials (RCTs) [48–69]. All the eligible studies used static stretching, and all the protocols targeted the lower limbs.

### Risk of Bias Within Studies

**Fig. 2:**
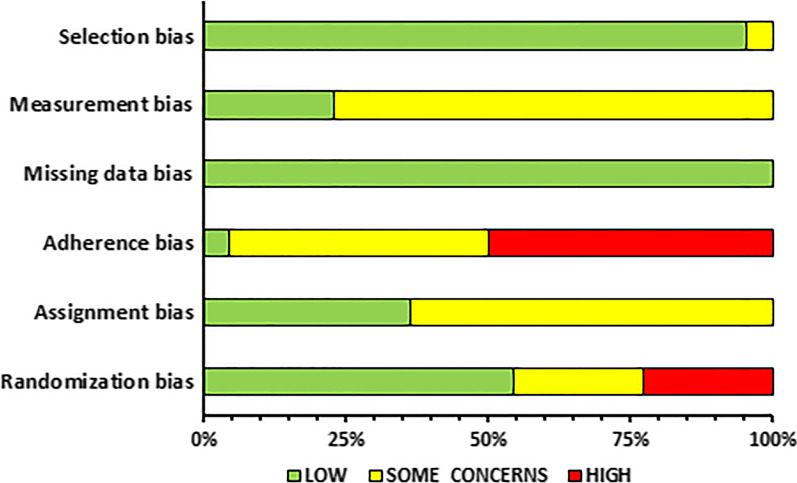
Summary of risk of bias assessment for randomized controlled trials

**Fig. 3:**
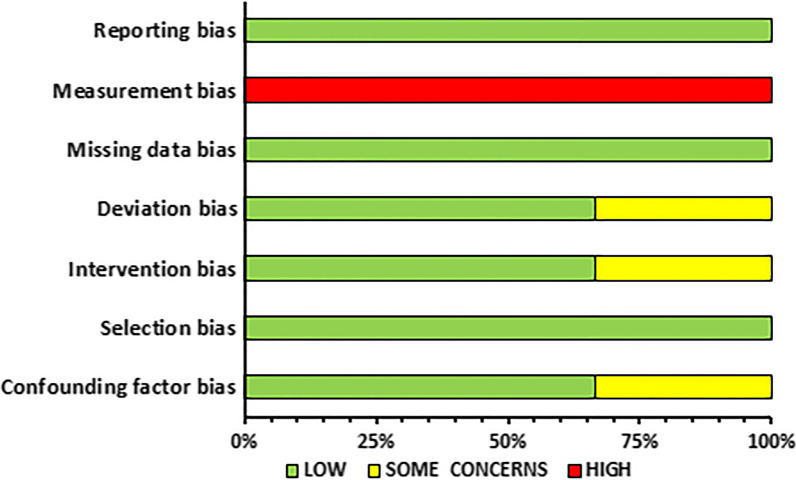
Summary of risk of bias assessment for controlled trials

A summary of the risk of bias assessment is illustrated in Figs. 2 and 3 for the RCTs and CTs, respectively. A detailed description of the risk of bias assessment for all the included studies in the current systematic review can be found in Additional file 1.

### Meta-analysis Outcomes

Primary outcomes were any assessments related to ROM changes, both short-term (> 2 weeks) and long-term (~ 9 months) in children and adolescents (≤ 11 vs. ≥ 12 years of age, respectively). These outcomes were used only if there were pre- and post-intervention assessments. Secondary outcomes included differences in ROM according to the stretching volume load (< 3600 s vs. ≥ 3600 s of total stretching duration). Subgroups analyses were performed according to age groups, as follows: child participants (≤ 11 years of age) following either a lower (< 3600 s) or a higher stretching volume load protocol (≥ 3600 s), and adolescents (≥ 12 years of age) following either a lower (< 3600 s) or a higher stretching volume load protocol (≥ 3600 s).

### Primary Outcomes

**Fig. 4:**
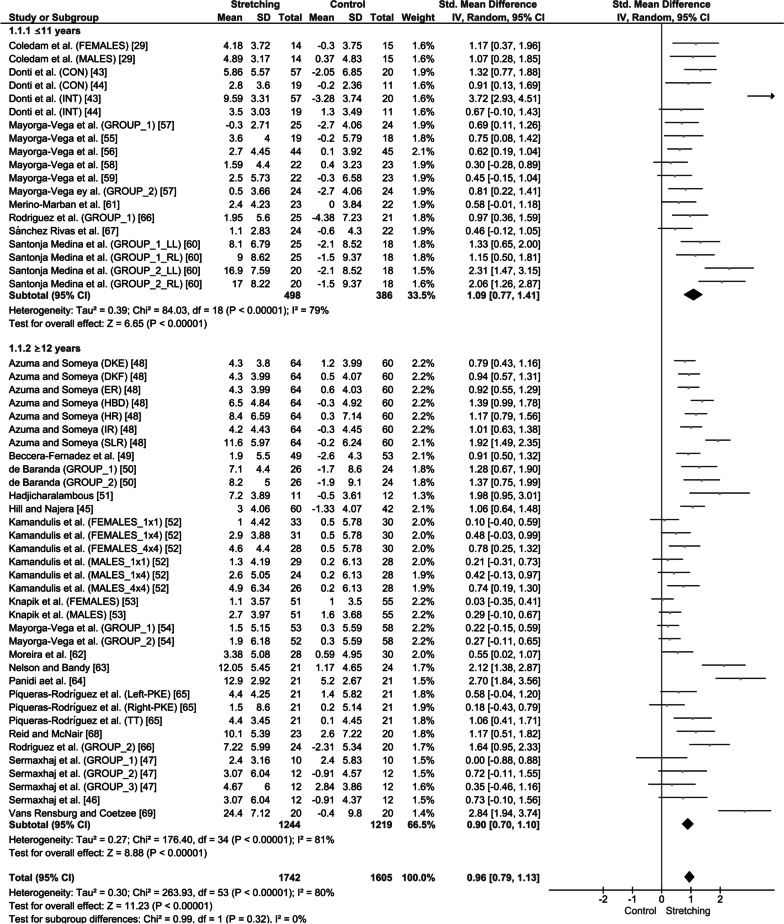
Effect of static stretching training on joint range of motion in children and adolescents. SD: standard deviation, 95% CI: confidence interval. *Note*: CON: continuous stretching; INT: intermittent stretching; LL: left leg; RL: right leg; DKE: dorsiflexion with knee extension; DKF: dorsiflexion with knee flexion; HR: hip rotation; ER: external rotation; IR: internal rotation; HBD: heel-to-buttocks distance; SLR: straight leg raise; SAR: sit and reach; TT: toe-touch; and PKE: passive knee extension

After all the participants had been analyzed together, it was found that stretching interventions were moderately effective in increasing ROM in the experimental groups compared with age-matched controls (SMD = 0.96; 95% CI = 0.79–1.13; *Z* = 11.23; *p* < 0.001; *I*^2^ = 80%; Fig. 4). In particular, the results showed that stretching was moderately effective in increasing ROM in children (SMD = 1.09; 95% CI = 0.77–1.41; *Z* = 6.65; *p* < 0.001; *I*^2^ = 79%; Fig. 4) and adolescents (SMD = 0.90; 95% CI = 0.70–1.10; *Z* = 8.88; *p* < 0.001; *I*^2^ = 81%; Fig. 4). However, no differences were found in ROM improvements between age groups (≤ 11 years of age vs. ≥ 12 years of age; SMD: 1.09 vs. 0.90, *p* = 0.32; *I*^2^ = 0%; Fig. 4).

### Secondary Outcomes

Out of the 54 entries analyzed, 27 had “low” total volume (i.e., < 3600 s) and 27 had “high” total volume (≥ 3600 s). The characteristics of stretching interventions in the two subgroups (“high” and “low” volume) differed only in the number of exercises per session (two exercises vs. six exercises, *p* ˂ 0.001), and in the duration of the intervention (8.2 ± 2.7 weeks vs. 18.4 ± 9.5 weeks, *p* < 0.001), while the number of sets, the duration of each stretching bout, and the frequency of training per week were similar (*p* ˃ 0.08) (see, published file: 10.6084/m9.figshare.17104640).

**Fig. 5:**
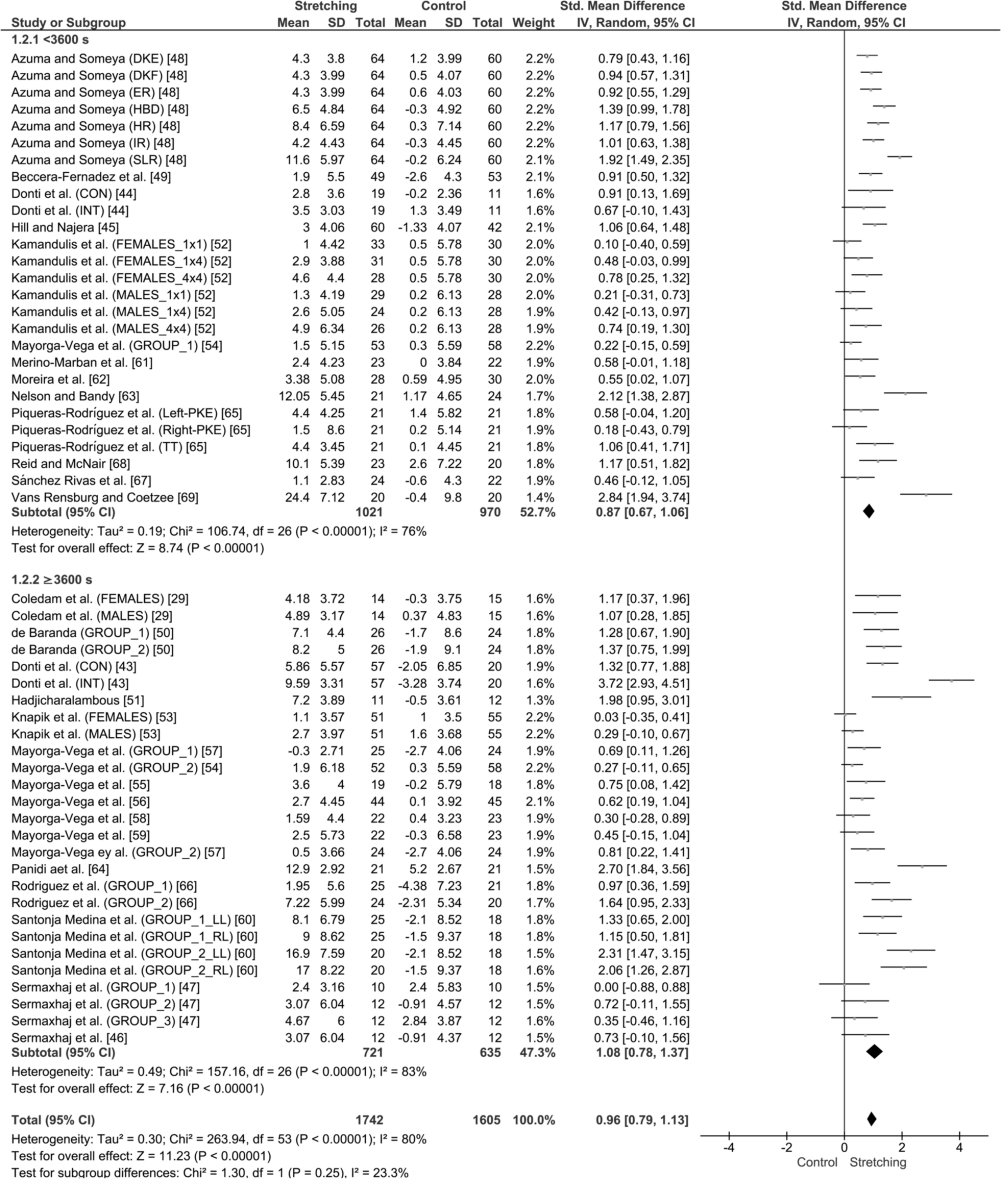
Effect of high and low stretching volume load on joint range of motion. SD: standard deviation, 95% CI: confidence interval. *Note*: CON: continuous stretching; INT: intermittent stretching; LL: left leg; RL: right leg; DKE: dorsiflexion with knee extension; DKF: dorsiflexion with knee flexion; HR: hip rotation; ER: external rotation; IR: internal rotation; HBD: heel-to-buttocks distance; SLR: straight leg raise; SAR: sit and reach; TT: toe-touch; and PKE: passive knee extension

After all the participants had been analyzed together, lower stretching volume loads (< 3600 s) increased ROM in the experimental groups compared with the age-matched controls (SMD = 0.87; 95% CI = 0.67–1.06; *Z* = 8.74; *p* < 0.001; *I*^2^ = 76%; Fig. 5) and the same was found for higher stretching volume loads (≥ 3600 s) (SMD = 1.08; 95% CI = 0.78–1.37; *Z* = 7.16; *p* < 0.001; *I*^2^ = 83%; Fig. 5). No differences were observed in ROM increases between higher and lower stretching volume loads when children and adolescents were analyzed together (SMD: 0.87 vs. 1.08; *p* = 0.25; *I*^2^ = 23.3%; Fig. 5).

### Subgroup Analyses: Age and Stretching Volume Interaction

**Fig. 6:**
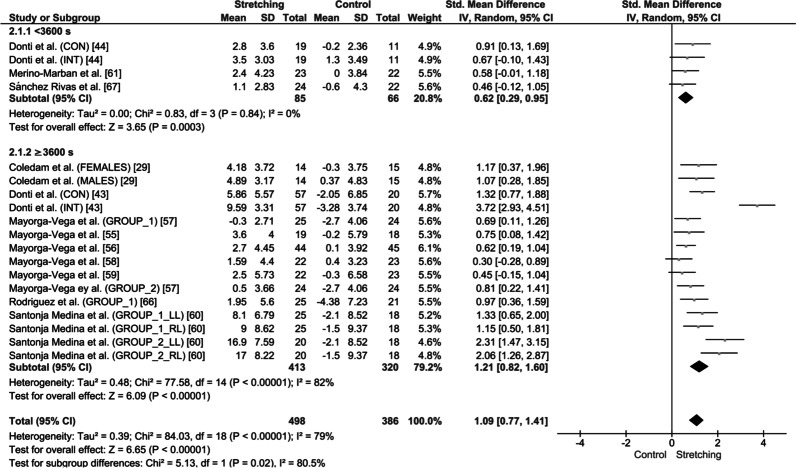
Effect of high and low stretching volume load on joint range of motion in children. SD: standard deviation, 95% CI: confidence interval. *Note*: CON: continuous stretching; INT: intermittent stretching; LL: left leg; and RL: right leg

Subgroup analyses in children (≤ 11 years of age) showed that both lower (< 3600 s) and higher (≥ 3600 s) stretching volume loads were effective in increasing ROM in the experimental groups compared with the controls (SMD = 1.09; 95% CI = 0.77–1.41; *Z* = 6.65; *p* < 0.001; *I*^2^ = 79%; Fig. 6). However, higher stretching volume loads were more effective in increasing ROM during childhood (SMD = 1.21; 95% CI = 0.82–1.60; *Z* = 6.09; *p* < 0.001; *I*^2^ = 82%; Fig. 6) compared with lower stretching volume loads (SMD = 0.62; 95% CI = 0.29–0.95; *Z* = 3.65; *p* = 0.0003; *I*^2^ = 0%; Fig. 6; SMD: 0.62 vs. 1.21, subgroup difference: *p* = 0.02; *I*^2^ = 80.5%; Fig. 6).

**Fig. 7:**
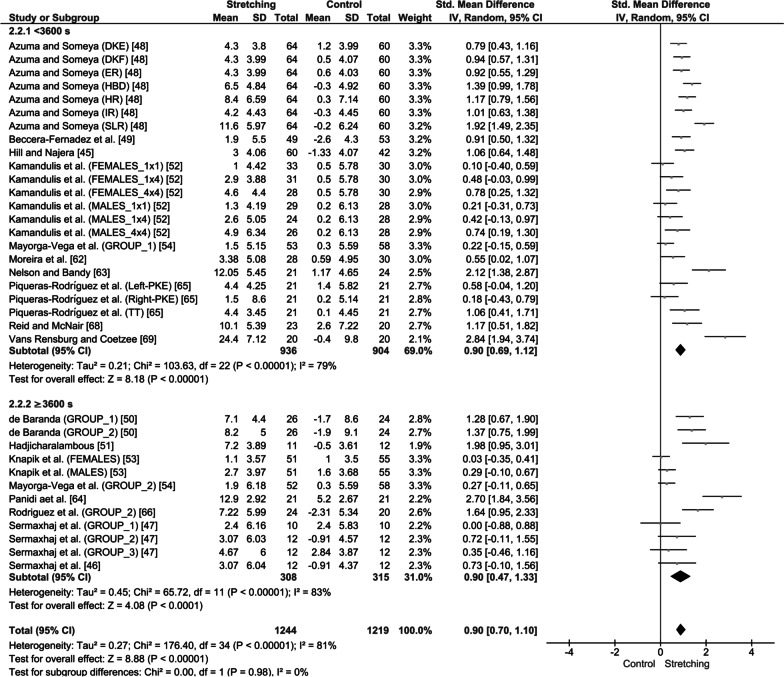
Effect of high and low stretching volume load on joint range of motion in adolescents. SD: standard deviation, 95% CI: Confidence Interval. *Note*: LL: left leg; RL: right leg; DKE: dorsiflexion with knee extension; DKF: dorsiflexion with knee flexion; HR: hip rotation; ER: external rotation; IR: internal rotation; HBD: heel-to-buttocks distance; SLR: straight leg raise; TT: toe-touch; and PKE: passive knee extension

Subgroup analyses in adolescents (≥ 12 years of age) showed that both stretching volume loads, i.e., lower (< 3600 s) and higher (≥ 3600 s), were effective in increasing ROM in the experimental groups compared with the controls (SMD = 0.90; 95% CI = 0.70–1.10; *Z* = 8.88; *p* < 0.001; *I*^2^ = 81%; Fig. 7). Higher stretching volume loads increased ROM during adolescence (SMD = 0.90; 95% CI = 0.47–1.33; *Z* = 4.08; *p* < 0.001; *I*^2^ = 83%; Fig. 7), and the same was found for lower stretching volume loads (SMD = 0.90; 95% CI = 0.69–1.12; *Z* = 8.18; *p* < 0.001; *I*^2^ = 79%; Fig. 7). No differences were found in ROM increases in adolescents between the two stretching volume loads (SMD = 0.90 vs. 0.90; subgroup difference: *p* = 0.98; *I*^2^ = 0%; Fig. 7).

### Confidence in Cumulative Evidence

Confidence in the cumulative evidence is equivalent to the quality of the evidence [35]. GRADE assessments are presented in Additional file 1. For randomized controlled trials, GRADE starts by assuming high quality, which can be downgraded according to five dimensions (risk of bias, inconsistency of results, indirectness, imprecision, and publication bias) [35, 36]. In this study, randomized controlled trials and controlled trials were included and GRADE thus started assuming moderate quality. The quality of evidence was not downgraded for risk of bias but was downgraded due to inconsistency of the results (one level) and indirectness (one level). For GRADE analysis, the following evaluation components were adopted to higher quality (large effect, dose–response, and confounding). Overall, the analysis showed that we can be moderately confident in the effect estimates. This implies that the true effect is likely to be close to the estimate of the effect. Visual inspection of the funnel plot implied no publication bias (Additional file 2: Fig. S1).

## Discussion

The aim of this systematic review and meta-analysis was to examine whether there is a difference in the effect of stretching training on flexibility during childhood and adolescence. The main meta-analysis, which included 28 studies and 54 effect sizes, indicated an increase in joint ROM after training in both children and adolescents with a medium magnitude of change (SMD = 0.96, *p* < 0.001), but no difference between children and adolescents when the effect of stretching volume load was not considered. However, the subgroup analyses showed that higher stretching volume loads result in larger ROM gains only during childhood and not in adolescence.

The main meta-analysis showed an equal increase in ROM in children (6–11 years of age) and adolescents (12–18 years of age), following stretching training. This finding appears to contradict the current suggestions in the pediatric literature regarding a “window of opportunity” for flexibility, i.e., an age range where training responses are maximized [3, 70]. Consequently, it has been suggested that if appropriate training is not performed during this “window,” maximum potential may not be reached [7]. The long-term athlete development model and the youth development model have suggested that middle childhood serves as an important time frame for flexibility development because it incorporates a period that has been termed “critical” for ROM enhancement [4, 8]. Although this suggestion may provide coaches and clinicians with a valuable insight into the components of a successful athletic development program, there is still no conclusive evidence to support this suggestion [1]. This is because evidence regarding ROM improvement following stretching training in children and adolescents is limited and inconsistent [71, 72], despite the fact that flexibility in young athletes is often associated with a higher performance, at least in sports such as gymnastics, swimming, and dance. The results of the current meta-analysis show that flexibility can be developed throughout childhood and adolescence, and there does not appear to be an effect of age on ROM development, at least for the training periods examined in the current systematic review (2–9 months). Along this line, Lloyd et al. [3] recently suggested that the concept of a “window of opportunity” is questionable and that most fitness components are trainable throughout childhood and adolescence, while training should not be considered as more effective in certain ages.

However, the subgroup analyses revealed a very interesting finding, i.e., that higher stretching volume loads result in larger ROM gains only in children and not in adolescents (Fig. 6). In contrast with the lack of difference in ROM improvements between children and adolescents, the interaction of age and stretching volume load seems to suggest that there may be indeed a “window of opportunity” during childhood for flexibility development, provided that the stretching volume load is more than 3600 s. It should be noted that the importance of flexibility is sport specific, and in sports such as gymnastics and dance, athletes are required to perform technical elements requiring large ROM from a very young age (7–9 years old) [72]. Therefore, if it is important to have a large joint ROM, then higher stretching volume loads could be successfully implemented during childhood. This finding warrants further investigation, because of the small number of studies implementing low-volume stretching protocols (i.e., lower than 3600 s) in children. Nevertheless, it was shown that, in childhood, higher training volumes can induce larger ROM gains, a finding possibly associated with the increased pliability and reduced musculotendinous stiffness observed during this period of development which may enable greater ROM to be attained [19]. A recent study found that the greater ankle dorsiflexion in the stretched compared with the control leg after 12 weeks of high-volume stretching training was accompanied by a concomitant increase in resting fascicle length of gastrocnemius medialis, greater fascicle elongation of gastrocnemius medialis and lateralis, and larger increases in gastrocnemius cross-sectional area in female adolescent athletes [64]. There is, however, a paucity of studies that have examined the association between joint ROM and muscle morphology, as well as other factors (i.e., growth, age, sex, training status, and type of joint/muscle examined) affecting flexibility at different developmental ages.

On the other hand, the subgroup analyses showed that in adolescence, higher and lower stretching volume loads both induce similar increases in ROM. The mechanisms associated with the response of children and adolescents to high-volume stretching have not yet been studied. Growth, maturation, muscle and tendon morphology, and neurophysiological differences between children and adolescents may underpin this response [19, 73–75]. During puberty, the growth of bones is faster than that of muscles, which can result in reduced muscle–tendon extensibility in postural and biarticular muscles, and substantial limitations on ROM [76–78]. In addition, the rise of hormone levels associated with puberty (e.g., testosterone) [79] may affect tendon stiffness and consequently ROM, at least in boys [80]. Since levels of flexibility tend to temporarily plateau or even decrease at the time of the adolescent growth spurt [81], the results of this meta-analysis suggest that higher stretching volume loads may not result in larger ROM gains at this age range. This finding is important because it suggests that the maintenance of the previously acquired levels of flexibility should be the training focus in adolescents for future athletic development [15].

The cutoff value for the stretching volume load in this systematic review (i.e., 3600 s), was determined by calculating the total stretching duration of 10 weeks of training, including three sessions per week, and two sets of two exercises performed for 30 s each. These stretching characteristics are commonly used in sports practice [41]. It should be noted that the two subgroups (“high” and “low” volume load) differed only in the number of exercises per session (two exercises vs. six exercises, *p* < 0.001), and in the duration of the intervention (8.2 ± 2.7 weeks vs. 18.4 ± 9.5 weeks, *p* < 0.001), while the number of sets and the frequency of training per week were similar. Thus, the sixfold difference in the mean stretching volume between the two subgroups (2062  vs. 12436 s) (see dataset file/10.6084/m9.figshare.17104640) was mainly due to the number of exercises per session and the training duration in weeks. The more than twofold training duration of the studies in the “high” subgroup (8.2 weeks vs. 18.4 weeks) may indicate that flexibility is a fitness component that is improved slowly, possibly due to the morphological adaptations that require more time to develop [64]. Although some flexibility gains may be noticed following only a few weeks of training, the large ROM adaptations observed in certain sports such as gymnastics and dance may need several months or even years to occur [43]. In this respect, more evidence is needed regarding the effects of long-term stretching protocols applied throughout childhood and adolescence, which could be a suggestion for future studies. Furthermore, it would be interesting to compare the effects of other types of training, such as strength and eccentric exercises, on ROM at developmental ages [82, 83].

To the best of the authors’ knowledge, this is the first systematic review and meta-analysis to have examined flexibility development during childhood and adolescence despite the importance of flexibility for young athletes. In this systematic review, a robust methodology was implemented [84–86], together with well-established tools to assess the quality of the included studies [87]. As indicated by the GRADE analysis, the findings of this meta-analysis are based on studies with a moderate quality of evidence, and thus, we are confident that the true effect is likely to be close to the estimate of the effect. In terms of population, a large sample of children (*n* = 652) and adolescents (*n* = 1284) was included in this meta-analysis, and thus, generalization of the findings to the respective populations is possible.

### Limitations

One limitation is that in this systematic review no comparisons were made between male and female participants because the studies including both males and females reported collective values for both sexes, with the exception of three studies [29, 52, 53]. Furthermore, no comparisons between athletic and non-athletic populations were performed because in the studies involving primary or secondary school students, extracurricular activities (e.g., sport participation) were not controlled for or were not reported. Finally, most of the included studies examined the hip joint (22 out of 28 studies, see 10.6084/m9.figshare.17104640), while there is a sparsity of information regarding upper limb flexibility.

## Conclusions

In conclusion, this meta-analysis indicated that systematic stretching training increases ROM during both childhood and adolescence. This may initially suggest that a “window of opportunity” for flexibility development during childhood which has been widely suggested in the literature is not evident, and flexibility can be developed throughout childhood and adolescence. However, the subgroup analyses showed that higher stretching volume loads result in larger ROM gains only in children and not in adolescents, thus suggesting that the interaction of age and stretching volume load may create a “window of opportunity” during childhood for flexibility development, provided that the stretching volume load is more than 3600 s. In contrast, the lack of a stretching volume load effect in adolescents may be due to the faster linear growth of bones compared with muscles, which may reduce muscle–tendon extensibility in postural and biarticular muscles and induce substantial limitations on ROM [76–78]. Thus, during adolescence, flexibility training seems to be independent of stretching volume load. It should be noted that these findings are based on limited evidence from the subgroup analyses, so that future randomized studies examining the effect of different stretching protocols on flexibility enhancement at different stages of development as well as on the factors associated with flexibility in young athletic and non-athletic populations are needed.

## Supplementary Information

**Additional file 1**. Search algorithm, risk of bias assessment for randomized controlled trials and control trials, Grading of Recommendations, Assessment, Development and Evaluations (GRADE) analysis and Preferred Reporting Items for Systematic Reviews and Meta-Analyses (PRISMA) checklist.

**Additional file 2**. Funnel plot for the meta-analysis of the effects of static stretching training on range of motion.

## Data Availability

The datasets generated and/or analyzed during the current study are available in the Figshare data repository file (10.6084/m9.figshare.17104640).

## Abbreviations

ROM: Range of motion
GRADE: Grading of recommendations: assessment: development and evaluations
RoB: Risk of bias
CTs: Controlled trials
RCTs: Randomized controlled trials
SMD: Standardized mean difference
CI: Confidence interval
I^2^: I^2^ statistic describes the percentage of variation across studies that is due to heterogeneity rather than chance
