# Supplementary figures and images for: Is There a “Window of Opportunity” for Flexibility Development in Youth? A Systematic Review with Meta-analysis
Source: Sports Med Open. 2022 Jul 6;8:88. doi: 10.1186/s40798-022-00476-1 (PMC9259532; doi:10.1186/s40798-022-00476-1)

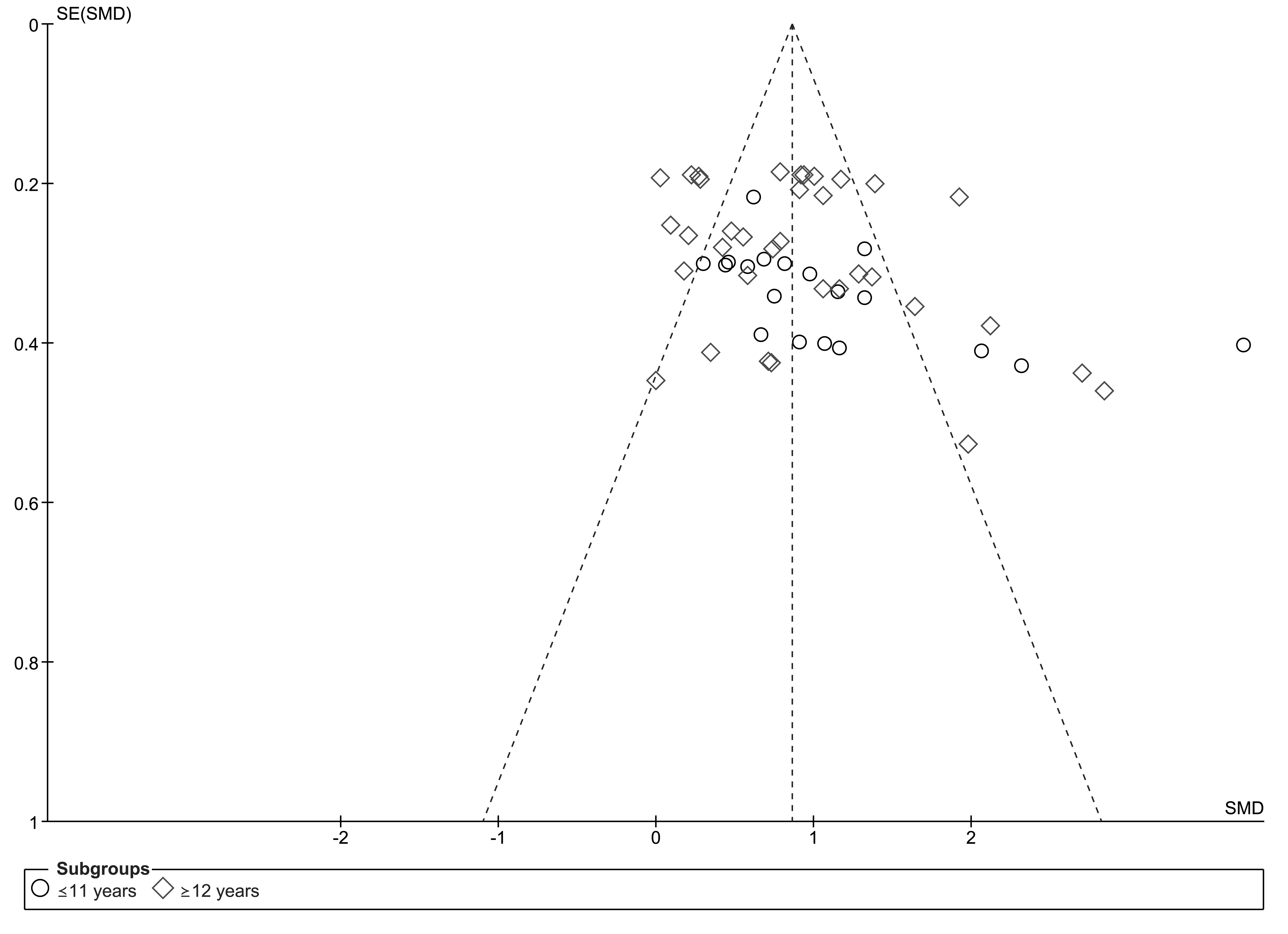

Supplement: Supplementary file 2 — Additional file 2. Funnel plot for the meta-analysis of the effects of static stretching training on range of motion. [file 40798_2022_476_MOESM2_ESM.tif]
